# Sex-Specific Protection of Endothelial Function after Vascular Ischemia/Reperfusion Injury by the Senomorphic Agent Ruxolitinib

**DOI:** 10.3390/ijms241411727

**Published:** 2023-07-21

**Authors:** Lars Saemann, Paula Naujoks, Lotta Hartrumpf, Sabine Pohl, Andreas Simm, Gábor Szabó

**Affiliations:** 1 Department of Cardiac Surgery, University Hospital Halle, 06120 Halle, Germany; 2 Department of Cardiac Surgery, Heidelberg University Hospital, 69120 Heidelberg, Germany

**Keywords:** ruxolitinib, senescence, ageing, ischemia/reperfusion injury, senotherapy, senomorphics, endothelium, senescence-associated secretory phenotype (SASP), proinflammatory cytokines, JAK, STAT

## Abstract

Ischemia/reperfusion (I/R)-induced endothelial dysfunction occurs in various cardiovascular disorders. I/R injury is partially driven by the release of cytokines. Known for its use in senotherapy, the JAK inhibitor ruxolitinib is able to block the release of cytokines. We investigated the effect of ruxolitinib on the cytokine release and endothelial-dependent vasorelaxation in an in vitro model of I/R. Aortic segments of C57BL/6J mice (N = 12/group) were divided into three groups: control, in vitro I/R (I/R group), and in vitro I/R with ruxolitinib during ischemic incubation (I/R+Ruxo group). We determined cytokine expression. In organ bath chambers, we investigated the maximal endothelial-dependent relaxation to acetylcholine (R_max_ACh) and maximal endothelial-independent relaxation to sodium-nitroprusside (R_max_SNP). R_max_ACh was decreased in I/R compared to the control (83.6 ± 2.4 vs. 48.6 ± 3.4%; *p* < 0.05) and I/R+Ruxo (74.4 ± 2.6 vs. 48.6 ± 3.4%; *p* < 0.05). R_max_SNP was comparable between all groups. IL-10 was detectable only in I/R+Ruxo. CXCL5, CCL2, CCL3, CCL8, CCL11, ICAM-1, IL-1α, IL-7, TNF-α, and G-CSF were decreased or not detectable in I/R+Ruxo. In I/R+Ruxo, ICAM-1 was reduced in rings only from male mice. Treatment of the aorta from mice during in vitro ischemia with the senomorphic agent ruxolitinib reduces cytokine release and protects the endothelium from I/R-mediated dysfunction.

## 1. Introduction

The vascular endothelium lines the entire circulatory system. It is characterized by various functions, such as regulating vascular tone to adjust the blood flow through vessels and preventing thrombosis and inflammation. Senescence and ischemia/reperfusion (I/R) injury promotes endothelial dysfunction [1,2]. I/R-induced endothelial dysfunction occurs in various cardiovascular disorders and treatment options, such as cardiopulmonary collapse and resuscitation, myocardial infarction and percutaneous coronary intervention (PCI) or coronary artery bypass grafting (CABG), and end-stage organ failure and organ transplantation [3,4]. Vascular I/R injury and endothelial dysfunction have immediate effects but may persist long-term and commonly lead to decreased short- and long-term outcomes [3].

I/R injury consists of two phases. The first phase is characterized by ischemia, anaerobic metabolism, and acidosis [2]. The second phase is characterized by reperfusion, reoxygenation, and the formation and release of multiple mediators and molecules, such as reactive oxygen species (ROS). Oxidative stress induces endothelial dysfunction and local inflammatory processes [2,5]. Furthermore, I/R injury is partially driven by mediators such as cytokines, chemokines, and growth factors, independently of age. 

The senomorphic agent ruxolitinib is a Janus kinase (JAK 1/2) inhibitor [6]. By replacing ATP on the catalytic site of the JAK tyrosine kinase, ruxolitinib inhibits the activation of the pathway [7]. Thus, binding cytokines cannot trigger the activation of the JAK, which would result in the phosphorylation of the signal transducer and activator of transcription (STAT). The aggregation of JAK and STAT would trigger stimulation and release of other cytokines such as G-CSF, IL3, IL-5, or IL-7 [7,8]. Due to the ability to block the release of several cytokines, ruxolitinib has also been used as a senotherapeutic drug [9]. Senescent cells accumulate with age in tissues and release various mediators summarized as senescence-associated secretory phenotype (SASP). Senescent cells also exist in a low number in the tissue of young animals. Even when the SASP is released from the minority of cells in a tissue, it has an effect on the neighboring cells and releases their viability. Furthermore, senotherapy is also efficient in young mice [10].

Thus, we investigated the effect of ruxolitinib on endothelial-dependent vasorelaxation and aortic cytokine expression in an in vitro model of vascular I/R using aortas from mice. 

## 2. Results

### 2.1. Vascular Contractility 

**Figure 1:**
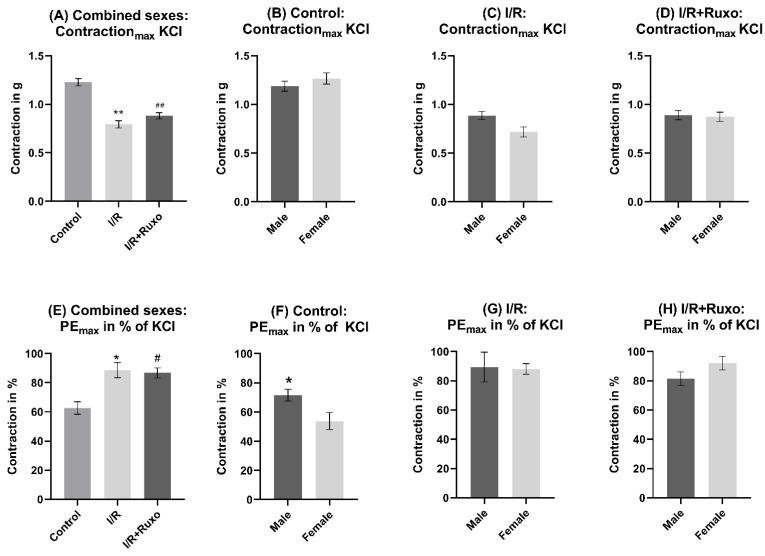
Maximal vascular contractility, combined sexes (**A**,**E**), and separated sexes (**B**–**D**,**F**–**H**). (**A**) Maximal contraction after KCl administration in (**B**) the control group, (**C**) I/R group, and (**D**) I/R+Ruxo group. (**E**) Maximal contraction after PE administration in % of KCl in (**F**) the control group, (**G**) I/R, and (**H**) I/R+Ruxo groups. * *p* < 0.05 and ** *p* < 0.001: I/R vs. control. ^#^ *p* < 0.05 and ^##^ *p* < 0.001: I/R+Ruxo vs. control. I/R: ischemia/reperfusion. KCl: potassium chloride. PE: phenylephrine. Ruxo: ruxolitinib.

**Figure 2:**
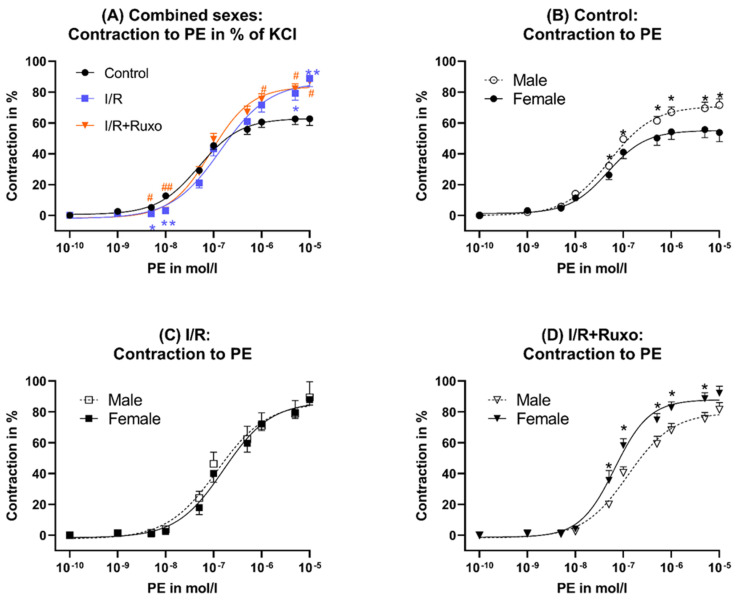
Vascular contractility, combined sexes (**A**) and separated sexes (**B**–**D**). (**A**) Contraction after PE administration in % of KCl; (**B**) control: contraction after PE administration in % of KCl; (**C**) I/R: contraction after PE administration in % of KCl; (**D**) I/R+Ruxo: contraction after PE administration in % of KCl; * *p* < 0.05 and ** *p* < 0.001: control vs. I/R, ^#^ *p* < 0.05 and ^##^ *p* < 0.001: control vs. I/R+Ruxo. I/R vs. I/R+Ruxo. I/R: ischemia/reperfusion. KCl: potassium chloride. PE: phenylephrine. Ruxo: ruxolitinib.

The maximal contractility after KCl administration decreased significantly in the I/R and I/R+Ruxo group compared to the control group (Figure 1A). However, both groups showed a significantly increased maximal contraction after PE administration compared to the control group (Figure 1E and Figure 2A). We could not detect sex-related differences in KCl-induced contraction (Figure 1B–D). The PE-induced contraction was significantly stronger in male mice compared to female mice in the control group (Figure 1F). In the I/R+Ruxo group, we demonstrated a significantly increased PE-induced contractility in female mice compared to male mice (Figure 2D). 

### 2.2. Endothelial-Dependent Relaxation

**Figure 3:**
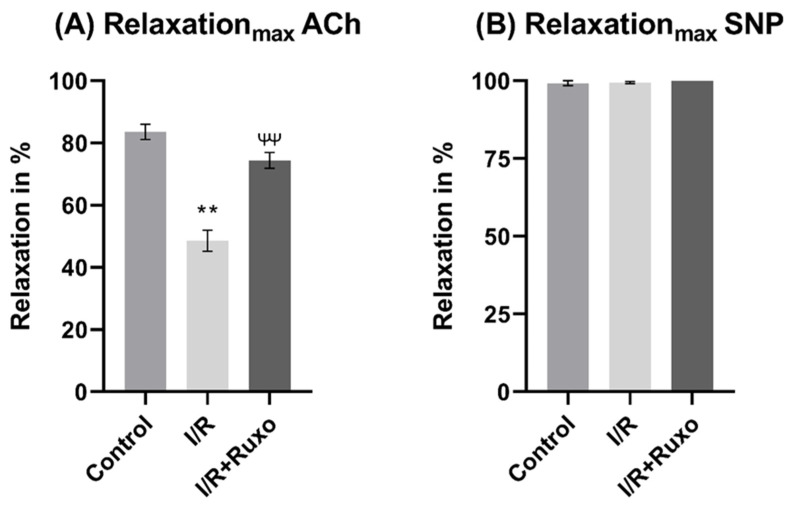
Maximal endothelial-dependent (ACh) and endothelial-independent (SNP) vascular relaxation. (**A**) Maximal relaxation after ACh administration; (**B**) maximal relaxation after SNP administration. ** *p* < 0.001: control vs. I/R; ^ψψ^ *p* < 0.001: I/R vs. I/R+Ruxo; ACh: acetylcholine. I/R: ischemia/reperfusion. SNP: sodium nitroprusside. Ruxo: ruxolitinib.

**Figure 4:**
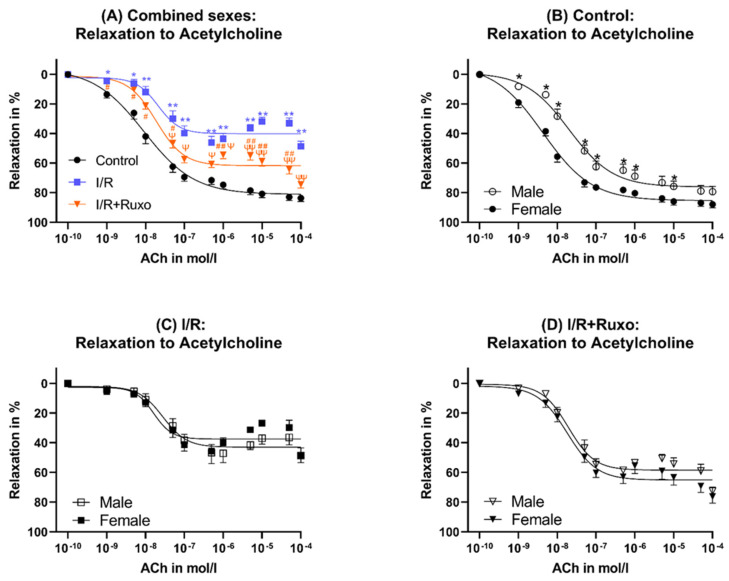
Endothelial-dependent relaxation, combined sexes (**A**) and separated sexes (**B**–**D**). (**A**) Relaxation after acetylcholine administration. * *p* < 0.05 and ** *p* < 0.001: compared to Control; ^ψ^ *p* < 0.05 and ^ψψ^ *p* < 0.001: compared to I/R; ^#^ *p* < 0.05 and ^##^ *p* < 0.001: compared to Control. ACh: acetylcholine. I/R: ischemia/reperfusion. (**B**) relaxation after acetylcholine administration. * *p* < 0.05. (**C**) I/R: relaxation after acetylcholine administration. (**D**) I/R+Ruxo: relaxation after acetylcholine administration. Ruxo: ruxolitinib.

In the I/R group, the ACh-mediated relaxation significantly decreased compared to both the control and I/R+Ruxo (Figure 3A). In the I/R+Ruxo group, the maximal relaxation to ACh significantly improved compared to I/R (Figure 3A). We could not detect sex-related differences regarding the maximal endothelial-dependent relaxation in the I/R or I/R+Ruxo groups (Figure 4C,D). 

### 2.3. Endothelial-Independent Relaxation

**Figure 5:**
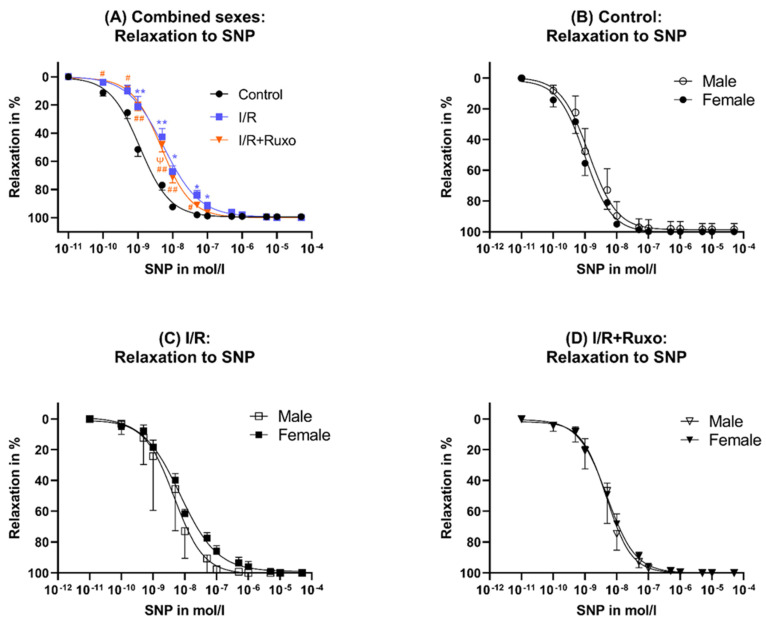
Endothelial-independent relaxation, combined sexes (**A**) and separated sexes (**B**–**D**). (**A**) Relaxation after SNP administration; * *p* < 0.05 and ** *p* < 0.001: compared to Control; ^ψ^ *p* < 0.05: compared to I/R; ^#^ *p* < 0.05 and ^##^ *p* < 0.001: compared to Control; (**B**) control: relaxation after SNP administration; (**C**) I/R: relaxation after SNP administration; (**D**) I/R+Ruxo: relaxation after SNP administration. I/R: ischemia/reperfusion. SNP: sodium nitroprusside. Ruxo: ruxolitinib.

The endothelial-independent relaxation was similar between the I/R and the I/R-Ruxo groups (Figure 5A). At lower concentrations, the SNP-induced vasorelaxation was significantly impaired in the I/R and I/R+Ruxo groups compared to the control group (Figure 5A). The maximal effect of SNP was comparable between all groups (Figure 3B). Our investigations showed no significant sex-related differences regarding SNP-mediated relaxation (Figure 5B–D).

### 2.4. Cytokine Release

**Figure 6:**
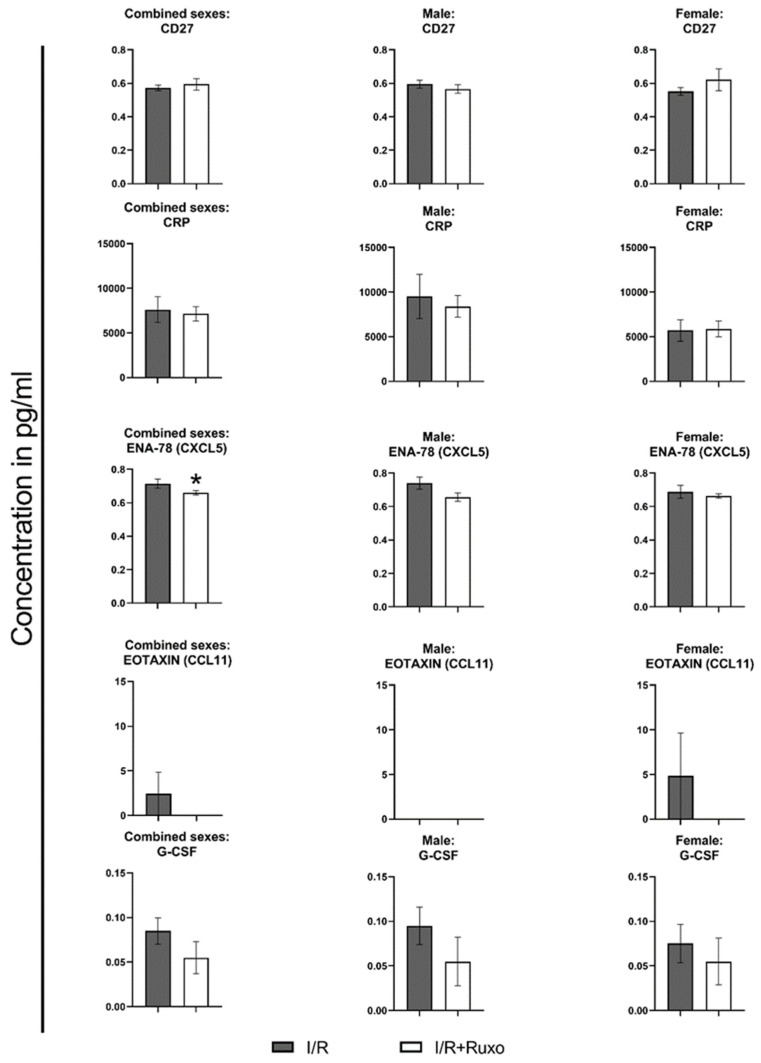
Cytokine release in 24 h incubation supernatant. We compared all detectable cytokines sex-wise. * *p* < 0.05: I/R vs. I/R+Ruxo. CCL: chemokine (c-c motif) ligand. CD: cluster of differentiation. CRP: C-reactive-protein. CXCL: chemokine (C-X-C motif) ligand. ENA: epithelial neutrophil-activating peptide. G-CSF: granulocyte-colony-stimulating factor. I/R: ischemia/reperfusion. Ruxo: ruxolitinib.

**Figure 7:**
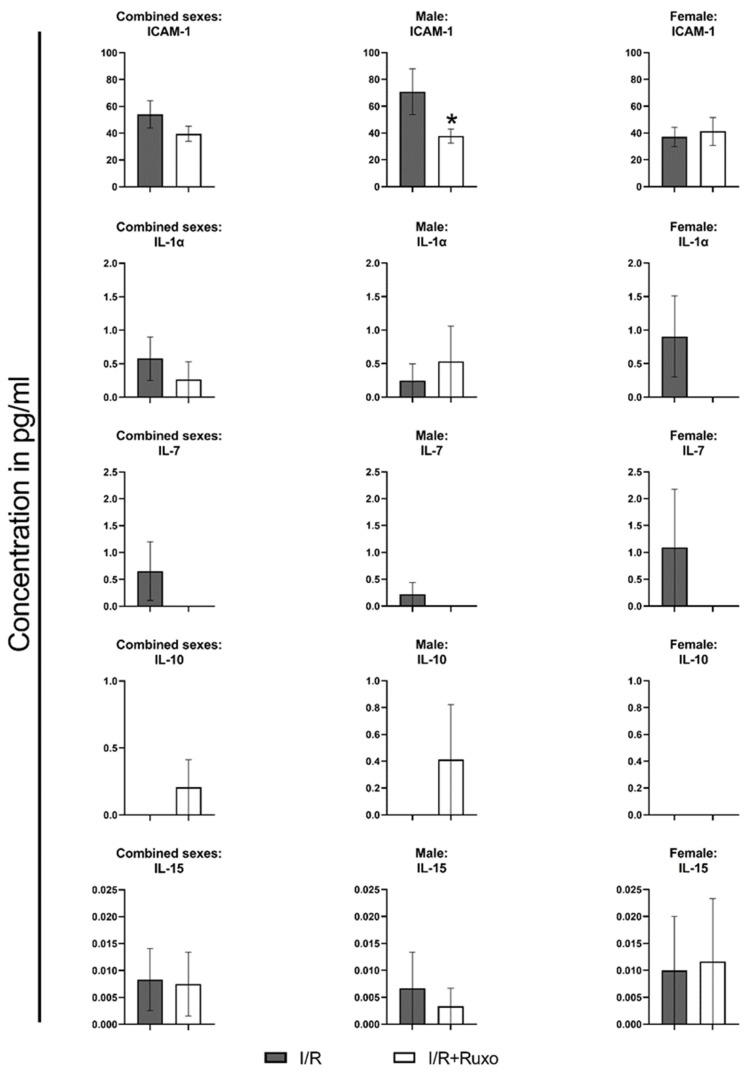
Cytokine release in 24 h incubation supernatant. We compared all detectable cytokines sex-wise. * *p* < 0.05: I/R vs. I/R+Ruxo. ICAM: intercellular adhesion molecule. IL: interleukin. I/R: ischemia/reperfusion. Ruxo: ruxolitinib.

**Figure 8:**
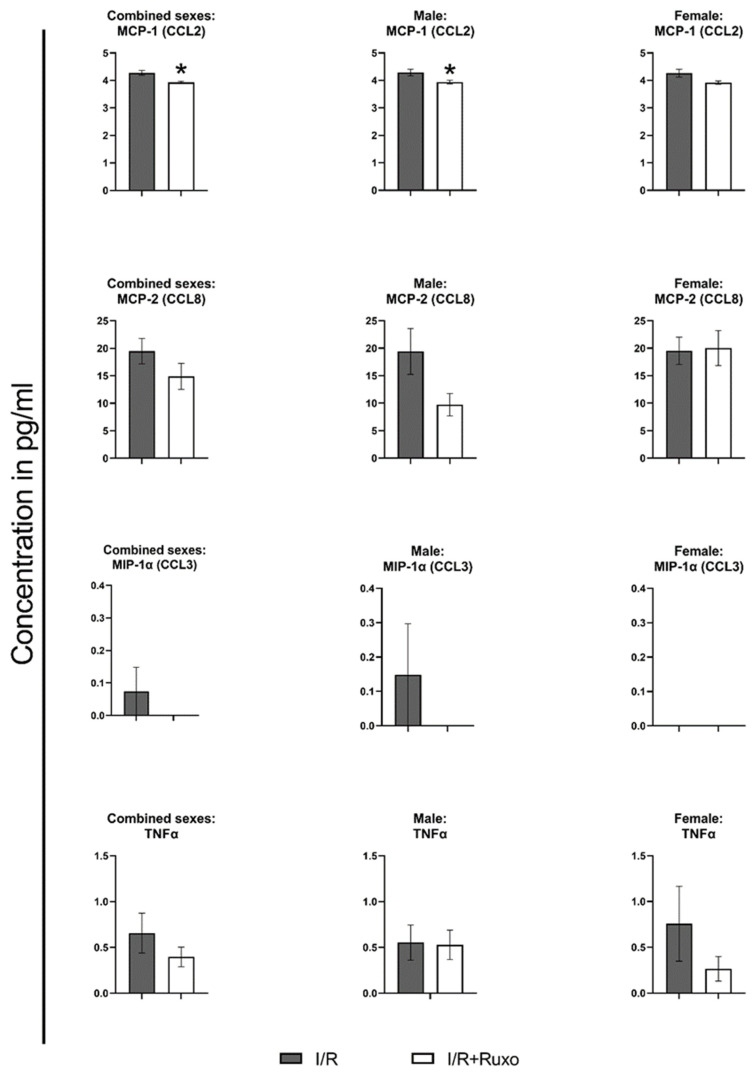
Cytokine release in 24 h incubation supernatant. We compared all detectable cytokines sex-wise. * *p* < 0.05: I/R vs. I/R+Ruxo. CCL: chemokine (c-c motif) ligand. I/R: ischemia/reperfusion. Ruxo: ruxolitinib. MCP: monocyte chemoattractant protein. MIP: macrophage inflammatory proteins. TNF: tumor necrosis factor.

We measured the release of 24 cytokines, chemokines, growth factors, and adhesion molecules in the supernate after 24 h ischemic incubation from both I/R and I/R+Ruxo groups. Ten mediators, namely, chemokine (CXC motif) ligand (CXCL)1, CXCL9, CXCL10, chemokine (c-c motif) ligand (CCL)5, interleukin (IL)-1β, IL-6, IL-13, granulocyte-macrophage colony-stimulating factor (GM-CSF), macrophage colony-stimulating factor (M-CSF), and vascular endothelial growth factor A (VEGF-A), were not detectable. IL-10 was detectable only in the I/R+Ruxo group. CD27, CRP, and IL-15 were released almost equally in both groups. Either CXCL5, CCL2, CCL3, CCL8, CCL11, intercellular adhesion molecule (ICAM)-1, IL-1α, IL-7, tumor necrosis factor (TNF)-α, and granulocyte-colony-stimulating factor (G-CSF) were released in lower amounts, or they were not detectable in the I/R+Ruxo group. We also focused on the sex-dependent differences between the two groups regarding cytokine release (Figure 6, Figure 7 and Figure 8). For most of the cytokines, the reduced secretion in ruxolitinib-treated aortic rings was noticeable in both rings from male and female mice. However, the expression of ICAM-1 (Figure 7) and CCL8 (Figure 8) decreased only after ruxolitinib treatment in aortic rings from male mice but not female mice. In addition, the reduced release of TNF-α after ruxolitinib treatment was only visible in the aortic rings of female but not male mice. 

## 3. Discussion

In the present work, we demonstrated that treatment of the aorta during in vitro ischemia with the JAK inhibitor ruxolitinib reduces the release of various cytokines, chemokines, and growth factors and protects the vascular endothelium from I/R-mediated injury in the aortas from young mice. The release of ischemia-mediated cytokines, chemokines, adhesion molecules, and growth factors in the aortas from young mice seems to be partially similar to but not congruent with the SASP-related cytokine release from old cells because ten cytokines of our customized panel were not detectable. However, concentrations below the detection level could also be caused by dilution due to the incubation volume of 150 µL. Thus, more cytokines might be detectable with incubation volumes below 150 µL. Nevertheless, incubating four aortic rings from mice at a volume below 150 µL in hypothermic conditions is technically challenging.

The PE-mediated contraction was similar in both sexes in the I/R group but different in the I/R+Ruxo group. This shows, at least during in vitro I/R, that ruxolitinib seems to protect the endothelial integrity or selective α1-adrenergic receptor mode of action in a sex-dependent manner. In the literature, the need to investigate the impact of sex on JAK inhibition has recently been described [11]. However, in the current state, nothing is known about the sex-specific effects of JAK inhibition by ruxolitinib.

Unlike PE, the effect on ACh-mediated vasorelaxation seems to be independent of sex. Our functional data showed that the relaxation in response to SNP, which depends on smooth muscle cells, revealed no difference between the I/R and I/R+Ruxo groups. Therefore, the decreased relaxation in the I/R group and improved relaxation in the I/R+Ruxo group in response to ACh were endothelial-dependent and not based on a change in the relaxation of smooth muscle cells. 

I/R injury is divided into decreased nutritional and oxidative supply and a second phase of re-established oxygenated perfusion. Reperfusion injury is marked by increased reactive oxygen species production and oxidative stress. This leads to endothelial dysfunction partially triggered by cytokine release [2,4,12]. It is known from the SASP that the expression of cytokines generally affects surrounding cells in a paracrine manner and thus reduces their viability and function [13,14]. The improved relaxation to ACh might have been caused by the reduced expression of CCL2, CCL8, CXCL5, and TNFα and possibly the increased release of IL-10, which all have receptors on endothelial cells [15,16,17,18,19]. 

The basal ICAM-1 expression is low in endothelial cells and can be enhanced by inflammatory cytokines [20]. Inflammatory cytokines were reduced by ruxolitinib in our study. Thus the reduced expression of proinflammatory cytokines could have been a reason for the reduced ICAM-1 expression [21,22]. However, the reduction of ICAM-1 was more pronounced in the aortic rings of male mice. CCL2 (MCP-1) and CCL8 (MCP-2) are CC chemokines that attract monocytes, T cells, mast cells, and basophils through their chemoattractant activity and thereby promote inflammatory processes [23]. This could lead to an even increased effect of ruxolitinib in an in vivo model of I/R. However, the release of CCL8 was decreased predominantly in the aortic rings of male mice. This could be one of the different potential reasons for the sex-dependent difference in PE-mediated contraction (Figure 2D). The secretion of CXCL5 (ENA-78) is increased after ischemic or inflammatory conditions [24].

Consequently, the decreased release of CXCL5 in the I/R+Ruxo group of this project suggests that ruxolitinib protects the vascular endothelium from ischemia. TNFα, a common proinflammatory mediator, was also increased after aortic in vitro I/R in mice of both sexes and was reduced slightly by ruxolitinib only in aortic rings from female mice [25,26,27]. Considering this, the reduced expression of TNFα might have been one reason for the improved relaxation after ruxolitinib treatment. IL-10 is an anti-inflammatory cytokine that can be protective after I/R in various tissues [28]. However, IL-10 was released inconsistently after ruxolitinib treatment. Consequently, the involvement of IL-10 in the partial preservation of the endothelial-dependent relaxation remains unclear and would become more evident in larger series.

Functional deterioration after vascular ischemia and reperfusion is a multifactorial pathophysiological process. It includes inflammation by the expression of cytokines, chemokines, and leukocyte adhesion molecules, and the production of reactive oxygen species, cell swelling, apoptosis, and necrosis [2,29]. In the present project, we only investigated the inflammatory contribution during ischemia. This comprised the possible contribution of cytokine, chemokine, interleukin, cluster of differentiation, and adhesion molecule expression to the functional decline of the aortic segments. Nevertheless, other pathways might have been involved as well.

Sex-dependent differences regarding the susceptibility of males to an increased I/R injury are commonly based on a differing hormonal cycle, in particular estrogen release, in females [30]. The estrogen receptor α especially increases females’ sensitivity for I/R [31]. In our in vitro experiments, we observed some sex-dependent differences regarding cytokine release and PE-induced vasocontraction but not endothelial-dependent vasorelaxation. Thus, the complex interplay between cytokines, endothelial-dependent vasorelaxation, and sex and age needs further investigation. Most likely, when used in aortic segments from old mice in an I/R model, the impact of ruxolitinib on cytokine release could be broader because, at old age, the age-associated and the I/R-associated cytokine secretion might cumulate. However, the dosage of ruxolitinib might need to be adjusted when used in aged animals. The transport of the agent into the cell might be decreased on the one hand, but toxic concentrations could also be lower, and pharmacokinetics might be slower on the other hand [32]. 

The reduced I/R-associated endothelial damage of our study is in line with the results from Zhu et al., who report that using ruxolitinib ameliorates ischemic stroke injury in mice after middle cerebral artery occlusion [33]. Furthermore, Yang et al. report attenuation of renal I/R injury and Khashab et al. demonstrated a reduced testicular I/R injury by the JAK2 inhibitor tyrphostin AG490 [34,35].

Considering the results of this work, the use of ruxolitinib during expected ischemia is promising. Expected ischemia occurs in organ preservation for transplantation, cardioplegic cardiac arrest during cardiac surgery, or circulatory arrest during aortic arch replacement. Furthermore, the potential effects of ruxolitinib administration at the onset of reperfusion after ischemia requires further investigation. The most common clinical situations of ischemia in cardiovascular medicine could be percutaneous coronary intervention, coronary artery bypass grafting, cerebral thrombolysis, or resuscitation. In old age, severe cardiovascular events, even when treated adequately, may trigger frailty that many patients often cannot overcome. It was shown by other groups that the continuous treatment of old mice with ruxolitinib decreased frailty in old age [36]. This finding highlights that in old patients who have experienced a severe cardiovascular event, which is commonly associated with severe I/R injury, treatment with ruxolitinib from the onset of reperfusion and continuing long-term might be promising.

When ruxolitinib is given systemically, the potential effects of JAK inhibition should also be considered. 

This study is limited by the fact that we applied an in vitro model of I/R that indicates several endothelial protective effects of ruxolitinib during vascular ischemia. Nevertheless, in vivo models are still needed.

## 4. Materials and Methods

### 4.1. Animals and Anesthesia

The appropriate Institutional Ethical Committee for Animal Experimentation reviewed and approved this study. The animals received humane care. We used male and female C57BL/6J mice (Janvier, Le Genest-Saint-Isle, France) at 3–5 months of age and a body weight of 20–30 g for the experiments. We injected 200 µL (500 i.U.) of heparin intraperitoneally and anesthetized the mice rapidly with isoflurane. The mice were euthanized by cervical dislocation.

### 4.2. Harvesting and Preparation of the Aorta

After euthanization, we immediately opened the thoracic cavity by bilateral thoracotomy. Then we carefully exposed and carefully harvested the descending thoracic aorta using microsurgical instruments under microscopic vision. The harvested graft was immediately placed on cold carbogenized Krebs–Henseleit solution (KHS), followed by careful removal of all periadventitial fat and connective tissue under microscopic vision. Afterward, we cut the aortic graft into four rings of 2 mm in length. 

### 4.3. Groups and In Vitro Ischemia/Reperfusion Model

**Figure 9:**
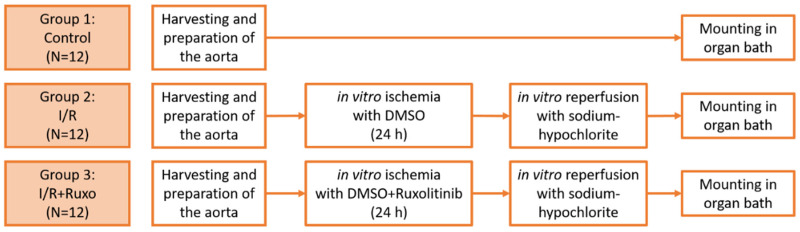
Groups and methodological process. I/R: ischemia/reperfusion. DMSO: dimethyl sulfoxide. Ruxo: ruxolitinib.

All groups consisted of equal numbers of male and female mice. Aortic rings remained untreated in a control group (N = 12) before further investigation (Figure 9). According to the groups, the aortic rings were stored for 24 h in 4 °C cold de-aired 0.9% saline solution with (I/R+Ruxo; N = 12) or without (I/R; N = 12) treatment with ruxolitinib (Selleckchem.com, Houston, TX, USA). To minimize the dilution of the aortic cytokine release, we used a volume of 147 µL of saline solution for ischemic incubation in microtubes with a total volume of 150 µL. Ruxolitinib was dissolved in 3 µL of DMSO (dimethyl sulfoxide) and added to the saline solution right before the beginning of the ischemic incubation to reach a final concentration of 500 nM ruxolitinib in the I/R+Ruxo group. The concentration was based on previous experiments including a stepwise adjustment of the concentration. In the I/R group, we only added an equal amount of DMSO to the saline solution. The microtubes were placed in a large de-aired tube filled with gaseous nitrogen. In another micro tube, we performed the whole ischemia procedure without vessel rings to measure the oxygen partial pressure at the end of the 24 h period to ensure ischemic conditions. After 24 h of in vitro ischemia, we mounted the aortic rings in organ bath chambers filled with 37 °C warm, carbogenized KHS. The supernatant of both groups was frozen in liquid nitrogen and stored at −80 °C for later cytokine quantification. We exposed the rings to 200 mM sodium hypochlorite for 30 min to mimic a reperfusion injury, according to the already published protocol [21,37]. In the control group, the rings were mounted immediately in organ bath chambers after preparation without ischemic storage or reperfusion injury. 

### 4.4. Organ Bath Functional Experiments

We mounted each aortic ring in a separate organ bath chamber (EMKA 4 Bath, EMKA Technologies S.A.S, Paris, France) filled with continuously carbogenized warm (37 °C) KHS. Initially, the aortic rings were equilibrated for 20 min at 0.1 g. Then, the tension of the vessel was periodically adjusted to 1.5 g over 60 min, including repeated washing steps with fresh KHS to remove potential metabolites. We exposed the vessel rings to 80 mM potassium chloride (KCl) to test the maximal receptor-independent contractility. When the aortic rings reached a stable plateau, we washed the organ bath chambers with fresh KHS and readjusted the tension to 1.5 g. With a gradually increasing concentration of the α-adrenergic receptor agonist phenylephrine (PE) (10^−9^–10^−5^ M), we induced vasoconstriction followed by gradually increasing concentrations of acetylcholine (Ach) to test the endothelial-dependent vasorelaxation. To investigate endothelial-independent relaxation, we induced one further contraction using a single dose of PE (10^−7^) followed by gradually increasing sodium nitroprusside (SNP) concentrations. 

### 4.5. Quantification of Cytokine Release

We performed a customized multiplex magnetic bead assay (ProcartaPlex Mouse and Rat Mix&Match Panels, Thermo Fisher Scientific, Waltham, MA, USA) with the incubation supernatant to quantify the aortic release of 20 cytokines, which were released during the ischemic incubation. In brief, we incubated 50 µL of the supernatant for 20 h with magnetic beads added to the plate. The remaining steps were conducted according to the manufacturer’s instructions, consisting of washing steps, the addition of a detection antibody, streptavidin-RPE, and a reading buffer.

### 4.6. Statistical Analysis

IBM SPSS Statistics for Windows (version 20.0, IBM Corp., Armonk, NY, USA) was used to perform the statistical analyses. Data were presented as mean ± standard error (SEM). We used Graph Pad Prism (Version 9, Graph Pad Software Inc., San Diego, CA, USA) to perform a nonlinear curve fit. The homogeneity of variance was tested with the Levene test. For multiple comparisons, we applied a one-way ANOVA with Tukey-adjusted *p*-values in the case of variance homogeneity and Games–Howell adjusted *p*-values in the case of variance inhomogeneity. We used a two-tailed unpaired classical *t*-test in variance homogeneity and a Welch *t*-test in variance inhomogeneity to compare cytokine expression and any sex-related differences. However, to emphasize the effects of sex-matching and -unmatching, we analyzed both combined sexes and separated sexes. A *p* < 0.05 was considered statistically significant.

## 5. Conclusions

In vitro I/R injury in aortic rings from young mice induces a cytokine release that is partially similar to the SASP-related cytokine release from old, senescent cells. Ruxolitinib during in vitro ischemia reduces the cytokine release with a sex-dependency for ICAM, CCL8, and potentially TNFα. On the functional level, ruxolitinib protects both the endothelial integrity and the endothelial-dependent relaxation from I/R-mediated damage. However, only the endothelial integrity is protected in a sex-dependent manner. Further experiments, including the in vivo use of ruxolitinib in clinically relevant conditions of I/R, such as solid organ transplantation, myocardial revascularization, resuscitation, or cerebral thrombolysis, considering sex and age, should be performed.

## Data Availability

Available on request.

## Abbreviations

ACh	acetylcholine
CABG	coronary artery bypass grafting
CCL	CC-chemokine ligand
CD	cluster of differentiation
CRP	c-reactive protein
GRO	growth-regulated-oncogene
CXCL	chemokine (C-X-C motif) ligand
DMSO	dimethyl sulfoxide
ENA	epithelial neutrophil-activating peptide
G-CSF	granulocyte-colony-stimulating factor
GM-CSF	granulocyte-macrophage colony-stimulating factor
M-CSF	macrophage colony-stimulating factor
KCl	potassium chloride
KHS	Krebs–Henseleit solution
ICAM	intercellular adhesion molecule
IL	interleukin
IP	interferon-gamma-induced protein
JAK	Janus kinase
I/R	ischemia/reperfusion
MCP	monocyte chemoattractant protein
MIG	monokine induced by gamma-interferon
MIP	macrophage inflammatory proteins
PCI	percutaneous coronary intervention
PE	phenylephrine
ROS	reactive oxygen species
SASP	senescence-associated secretory phenotype
SC	senescence cell
SEM	standard error of the mean
SNP	sodium nitroprusside
TNFα	tumor necrosis factor α
VEGF-A	vascular endothelial growth factor A
